# Transcriptome Sequencing Reveals Tgf-β-Mediated Noncoding RNA Regulatory Mechanisms Involved in DNA Damage in the 661W Photoreceptor Cell Line

**DOI:** 10.3390/genes13112140

**Published:** 2022-11-17

**Authors:** Yuke Huang, Xi Chen, Zhigao Jiang, Qian Luo, Linxi Wan, Xiangtao Hou, Keming Yu, Jing Zhuang

**Affiliations:** 1 State Key Laboratory of Ophthalmology, Zhongshan Ophthalmic Center, Sun Yat-sen University, Guangdong Provincial Key Laboratory of Ophthalmology and Visual Science, Guangzhou 510060, China; 2 School of Life Sciences, Sun Yat-sen University, Guangzhou 510060, China

**Keywords:** photoreceptors, DNA damage and repair, Tgf-β, competing endogenous RNA (ceRNA), *Gm20559*, *miR-361-5p*, *guanylate-binding protein 7* (*Gbp7*), *2’-5’-oligoadenylate synthetase 2* (*Oas2*)

## Abstract

Transforming growth factor β (Tgf-β), a pleiotropic cytokine, can enhance DNA repair in various cells, including cancer cells and neurons. The noncoding regulatory system plays an important role in Tgf-β-mediated biological activities, whereas few studies have explored its role in DNA damage and repair. In this study, we suggested that Tgf-β improved while its inhibitor LSKL impaired DNA repair and cell viability in UV-irradiated 661W cells. Moreover, RNA-seq was carried out, and a total of 106 differentially expressed (DE)-mRNAs and 7 DE-lncRNAs were identified between UV/LSKL and UV/ctrl 661W cells. Gene ontology and Reactome analysis confirmed that the DE-mRNAs were enriched in multiple DNA damaged- and repair-related biological functions and pathways. We then constructed a ceRNA network that included 3 lncRNAs, 19 miRNAs, and 29 mRNAs with a bioinformatics prediction. Through RT-qPCR and further functional verification, 2 Tgf-β-mediated ceRNA axes (*Gm20559*-*miR-361-5p*-*Oas2/Gbp7*) were further identified. *Gm20559* knockout or *miR-361-5p* mimics markedly impaired DNA repair and cell viability in UV-irradiated 661W cells, which confirms the bioinformatics results. In summary, this study revealed that Tgf-β could reduce DNA damage in 661W cells, provided a Tgf-β-associated ceRNA network for DNA damage and repair, and suggested that the molecular signatures may be useful candidates as targets of treatment for photoreceptor pathology.

## 1. Introduction

Photoreceptor cells (PRs) are principal light-sensitive, terminally differentiated neurons in the outer retina. Malfunction or loss of PRs is involved in multiple blindness-causing and intractable diseases, such as retinitis pigmentosa (RP) [1] and age-related macular degeneration (AMD) [2]. Notably, due to their constant exposure to light and metabolically active characteristics, PRs are rather susceptible to oxidative stress and DNA damage, which act as important factors of pathogenesis of the aforementioned diseases [3,4,5]. As such, to improve vision retention and disease prognosis, we sought to determine the mechanism of DNA damage/repair regulation in PRs to promote proper DNA repair and subsequent cell survival.

Neurons are highly susceptible to DNA damage, including single-strand breaks (SSBs) and double-strand breaks (DSBs). SSBs are among the most common lesions and can arise directly from attack or indirectly during the repair of base and nucleotide damage [6]. Meanwhile, DSBs are routinely formed in neurons under normal conditions [7]. γ-H2AX is at the center of a repair signaling cascade initiated by DSBs, and its locus formation is now generally accepted as a quantitative marker of DSBs [8]. DSB repair includes homologous recombination (HR) and nonhomologous end joining (NHEJ) [9]. Terminally differentiated PRs lack DNA replication-dependent DNA repair ability such as HR [7]. Thus, NHEJ is the key pathway in DSB repair in PRs. DNA stability serves an important role in neural viability because improper repair or a failure to repair DNA breaks could lead to a “domino effect”, causing gene deletions, translocations and missegregation of large chromosome fragments, and even apoptosis. Therefore, it is urgent to explore DNA repair mechanisms in the protection of PRs.

Previous studies have indicated that Tgf-β can promote DNA repair through various mechanisms [10,11,12]. The Tgf-β isoforms (Tgf-β1, -β2, and -β3) are 25-kDa homodimeric polypeptides [13]. They are secreted pleiotropic factors that play critical roles in a wide variety of biological activities, including DNA damage and repair, cell proliferation, and migration [14]. Li et al. reported that upon DNA damage, ataxia telangiectasia mutated (ATM)-mediated Tgf-β activation helps with complete cell cycle arrest and efficient DNA damage repair [12]. It was proven that canonical Tgf-β signaling reduces γ-irradiation-induced γ-H2AX foci formation in lung epithelial cells [15]. Moreover, Tgf-β could enhance DNA repair and neuroprotective effects by inducing growth arrest and DNA damage protein 45b (Gadd45b) in retinal ganglion cells [11]. Furthermore, we previously found that Tgf-β1 expression was significantly reduced upon ultraviolet (UV) treatment in 661W cells, an immortalized cone photoreceptor cell line [6]. Meanwhile, the blockage of Tgf-β1 by LSKL induced a significant increase in γ-H2AX expression upon UV treatment. However, the downstream molecular mechanisms of Tgf-β remain largely unknown and require further study.

The noncoding regulatory system has been shown to play an important role in Tgf-β-mediated biological activities, including epithelial-mesenchymal transition, migration, and tumor metastasis [16,17]. However, there are currently no studies pertaining to the noncoding mechanisms of Tgf-β-mediated DNA damage/repair in PRs. LncRNAs and miRNAs are two common kinds of noncoding RNAs (ncRNAs). LncRNAs can compete with miRNAs, acting as molecular sponges, thereby upregulating corresponding mRNAs. With the idea that lncRNAs and mRNAs may compete with each other for miRNAs, the strategy of the use of “competing endogenous RNA” (ceRNA) was carried out [18]. The comprehensive posttranscriptional regulatory network formed by ceRNA activity has greatly broadened the understanding of the mechanisms underlying various biological activities and diseases. Therefore, establishing a ceRNA network could help us better understand the mechanisms by which Tgf-β promotes DNA repair in PRs.

To address the questions above, we first verified the effect of Tgf-β and its inhibitor LSKL on the DNA damage response. Then, we carried out RNA-seq to analyze the expression profiles of the UV-treated control and experimental (LSKL+UV) groups and identified differentially expressed (DE-) lncRNAs and DE-mRNAs. Comprehensive bioinformatic tools were utilized to further explore the functions and interactions of DE-mRNAs, including gene ontology (GO) terms and pathway enrichment analysis, protein-protein interaction (PPI) networks, and hub gene analysis. Finally, we established a ceRNA network through in silico prediction and experimental and functional verification, widening the understanding of the mechanisms by which Tgf-β promotes DNA repair in PRs and thereafter providing novel biomarkers for DNA damage and repair, as in retinal pathology.

## 2. Materials and Methods

### 2.1. Ethics Statement

This study was approved and monitored by the Institutional Animal Care and Use Committee of Zhongshan Ophthalmic Center (Permit Number: SYXK (YUE) 2018-0189, 2020-155) and strictly complied with the ARVO Statement for the Use of Animals in Ophthalmic and Vision Research. C57/BL6 mice were provided by the animal center of Sun Yat-sen University (Guangzhou, China). The mice were sacrificed with an intraperitoneal injection of 4% chloral hydrate (Sigma, St. Louis, MO, USA) before eye resection to minimize suffering.

### 2.2. 661W Cells Culture and Treatment

Mouse retinal 661W cells were purchased from ATCC (Manassas, VA, USA) and cultured in Dulbecco’s modified Eagle’s medium (DMEM, Gibco, CA, USA) supplemented with 10% fetal bovine serum (FBS; Gibco, Carlsbad, CA, USA). The cells were maintained at 37 °C in a humidified 5% CO2 incubator.

661W cells were divided into 5 groups: (1) UV group, in which a control vehicle was added before UV exposure (30 J/m^2^, 254 nm, irradiated for 40 s); (2) Tgf-β group, in which Tgf-β (Sinobiological, Beijing, China, final concentration at 1 ng/mL [19]) was added 1 h prior to UV exposure; (3) LSKL group, in which LSKL (MCE, HY-P0299, final concentration at 10 μg/mL) was added 24 h prior to UV exposure; (4) Tgf-β + LSKL, in which a pretreatment with Tgf-β for 1 h and LSKL for 24 h followed by UV exposure was added; and (5) Sham, in which there was no UV exposure. For UV exposure, the cell medium was removed and replaced with PBS, and after 40 s of UV radiation, the cells were reincubated with a fresh cell culture medium and allowed to repair in the dark.

### 2.3. Primary Retinal Neurons Culture and Treatment

Postnatal 1–3 days old C57/BL6 mice were sacrificed with an intraperitoneal injection of 4% chloral hydrate (Sigma-Aldrich, St. Louis, MO, USA). The eyes were then resected, and the retinas were separated. The separated retinas were incubated in a solution containing 0.125% trypsin for 15 min at 37 °C to dissociate the cells. Then, the trypsinization was terminated by adding DMEM (Gibco, Carlsbad, CA, USA) supplemented with 10% FBS (Gibco, Carlsbad, CA, USA). The cells were seeded on plates precoated with 0.01% poly-D-lysine (Sigma-Aldrich, St. Louis, MO, USA) at approximately 1 × 10^6^ cells/mL. After cultured for 24 h, the culture media was changed to neurobasal media.

Primary retinal cells were divided into 2 groups: (1) UV group, in which a control vehicle was added 24 h before UV exposure (30 J/m2, 254 nm, irradiated for 30 s) and (2) LSKL group, in which LSKL (MCE, HY-P0299, final concentration at 10 μg/mL) was added 24 h prior to UV exposure. For UV exposure, the cell medium was removed and replaced with PBS, and after 30 s of UV radiation, the cells were reincubated with a fresh cell culture medium and allowed to repair in the dark.

### 2.4. Immunofluorescence Assay

Cells were seeded on coverslips in six-well plates and treated with different procedures. At the 2 h mark after UV radiation, cells on coverslips were fixed with 4% paraformaldehyde (Leagene, Beijing, China) for 15 min at room temperature, permeabilized with 0.1% Triton X-100 (Sigma, St Louis, MO, USA) for 10 min, and blocked with 5% BSA for 1 h. Afterwards, 661W cells were incubated with anti-phospho-H2AX (Ser139) antibody (clone JBW301, Millipore, Billerica, MA, USA) diluted in 5% BSA (1:100) overnight at 4 °C. Primary retinal neurons were incubated with anti-phospho-H2AX (Ser139) antibody (1:100) and MAP2 (Boster, Wuhan, China) antibody (1:100) overnight at 4 °C. Cells were then washed three times for 10 min each in PBS. The 661W cells were incubated with Alexa Fluor 555 anti-rabbit lgG (Cell Signaling Technology, Inc.) diluted in 5% BSA (1:500) for 1 h at room temperature, and the primary retinal neurons were incubated with both Alexa Fluor 555 anti-rabbit lgG (1:500) and Alexa Fluor 488 anti-mouse lgG (1:500). The cells were then washed again three times for 10 min each with PBS. Nuclei was visualized with DAPI staining and mounted on slides. Images were obtained using a Zeiss Axio Imager.Z2 microscope. Cells with more than 10 foci were recognized as γ-H2AX positive. The percentages of γ-H2AX-positive cells among DAPI-positive 661W cells or MAP2-positive primary retinal neurons were determined in samples from at least three independent experiments. Images from 8 randomly selected fields were used for analysis in ImageJ software. At least 3000 cells were quantified.

### 2.5. Comet Assay

The alkaline comet assay was performed using the comet assay reagent kits (Trevigen, Gaithersburg, MD, USA, 4250–050-K) according to the manufacturer’s protocol. Briefly, 661W cells were washed with ice-cold PBS and suspended in cold PBS 30 min after UV radiation. Then, the cells were combined at 1 x 10^5^/mL with molten LMAgarose (at 37 °C) at a ratio of 1:10, and they were pipetted 50 μL to the CometSlide^TM^. The slides were placed at 4 °C in the dark for 30 min, and then the slides were incubated in 4 °C Lysis Solution overnight. The slides were then immersed in freshly prepared Alkaline Unwinding Solution for 20 min at room temperature. Then, the electrophoresis was performed with 4 °C Alkaline Electrophoresis Solution at 25 Volt for 20 min. The slides were immersed twice in dH2O for 5 min each and in 70% ethanol for 5 min. After the slides were placed at 37 °C for 15 min, 100 μL diluted Gel-Red (Beyotime Biotechnology, Shanghai, China) was added onto the gel area and stained for 30 min. Then, images were obtained using a Zeiss Axio Imager.Z2 microscope. The tail length and olive tail moment were calculated using comet assay software.

### 2.6. RNA-seq Analysis and Differential Expression Analysis

The 661W cells were pretreated with 10 μg/mL LSKL for 24 h and irradiated with UV (30 J/m^2^, 254 nm) for 40 s. The total RNA was extracted from the 661W cells with Tri Reagent (Sigma-Aldrich, St. Louis, MO, USA, T9424) 1 h after UV radiation. Sequencing was performed on an Illumina NovaSeq 6000 with paired-end 150 bp using a TruSeq SBS Kit v4-HS (Illumina, Inc, San Diego, CA, USA).

For gene differential expression analysis, we used R software and the R package biomaRT to annotate the obtained RNA-seq data (HT-seq counts) [20,21]. Afterward, differential mRNA and lncRNA expression was analyzed between the two groups using the R package edgeR with library sizes normalized by the median count ratios across transcripts [22,23]. The *p*-values were calculated based on the Poisson distribution and corrected with the Benjamini-Hochberg (BH) correction. A false discovery rate (FDR) < 0.05 and |log2FoldChange| > 1 were set as the criteria for DE-genes screening.

Volcano plots were generated with the R package ggplot2 to visualize gene expression differences [24], and a heatmap was also produced to illustrate the gene expression profile of DE-genes with the pheatmap package of R. The expression data matrix was row-normalized for each gene prior to the application of average linkage clustering.

### 2.7. Functional Enrichment and Protein-Protein Interaction Analysis

A GO enrichment analysis was carried out using the R clusterProfiler package in the mouse annotation database org.Mm.eg.db [25]. A pathway enrichment analysis based on the Reactome database was conducted with the R package ReactomePA. The *p*-values were calculated based on a hypergeometric distribution using the Benjamini-Hochberg (BH) method. The pathways with a corrected *p*-value < 0.05 and *q*-value < 0.2 were considered significantly enriched. The results were visualized using custom plotting functions, with DNA damage-related GO and Reactome results displayed.

Differentially expressed mRNAs (DE-mRNAs) were used to construct a protein-protein interaction (PPI) network with the STRING database (http://www.string-db.org, accessed on 10 February 2021) [26]. The minimum required interaction score was set as high confidence (>0.9), and the active interaction sources were chosen as follows: Textmining, Experiment, Database, and co-expression. The results were downloaded in the TSV format and imported into Cytoscape software for visualization and further analysis of the functional protein-protein interaction networks [27]. The cytoHubba plugin was used to analyze hub genes, and maximal clique centrality (MCC) was used for calculating [28].

### 2.8. Construction of the ceRNA Regulatory Network

The target miRNAs for DE-lncRNAs were predicted using the DIANA database (https://diana.e-ce.uth.gr/lncbasev3, accessed on 15 February 2021) [29], which includes miRNA-lncRNA interactions supported by either a computational prediction or experimental verification. The target miRNAs for DE-mRNAs were predicted using miRwalk3.0 (adjusted binding probability > 0.95) [30], Mirtarbase (http://mirtarbase.mbc.nctu.edu.tw/index.html, accessed on 15 February 2021), miRDB (http://www.mirdb.org/, accessed on 15 February 2021), and TargetScan (http://www.targetscan.org, accessed on 15 February 2021). The predicted results were processed in R, including intersection and filtration, and the ceRNA network was plotted with Cytoscape v3.6.0. [31].

### 2.9. Reverse Transcription-Polymerase Chain Reaction (RT-qPCR)

The total RNA was isolated with a TRIzol reagent (Invitrogen, Carlsbad, CA, USA) 1 h after UV radiation. The cDNA was synthesized from the total RNA with The PrimeScriptTM RTPCR kit (Takara, Dalian, China) to detect mRNA and lncRNA. Mature miRNAs were converted into cDNA using reverse transcription performed by the RevertAid First Strand cDNA Synthesis Kit (Thermo Scientific, Waltham, MA, USA) with miRNA-specific primers. The RT-qPCR reaction was performed in triplicate. Expression levels were measured with RT-qPCR with the SYBR Green system (Roche, Indianapolis, IN, USA). β-Actin served as a reference control. Each sample was analyzed in triplicate. The primer pairs used in this article are listed in Supplementary Table S1.

### 2.10. Cell Viability Assayed by Cell Counting Kit-8 (CCK8) Assay

The cell viability was assessed with a CCK8 assay (Biosharp, Guangzhou, China) according to the manufacturer’s protocol. Briefly, 5 × 10^3^ cells were seeded into a 96 well plate. A 10 μL CCK8 reagent was added to each well 6 h after UV radiation. The cells were then incubated for 2 h at 37 °C. After incubation, the optical density (OD) value at 450 nm was measured. A cell-free well with the same volume of DMEM was used as a blank. Cell viability was determined by the ratio of the OD value of a treated culture to that of an untreated control.

### 2.11. siRNAs and miRNA Mimics

The siRNAs and miRNA mimics were provided by Tsingke Biological Technology company (TsingKe, Beijing, China). Details of the sequences are shown in Supplementary Table S2.

### 2.12. Transfection

Cells were transfected with 50 nM siRNA or miRNA mimics for 48 h using Lipofectamine 3000 (Invitrogen, Carlsbad, CA, USA) according to the manufacturer’s protocol. The 661W cells were 70% confluent at the time of transfection and transfected in Opti-MEM (Gibco, Carlsbad, CA, USA). The serum-free medium was changed to DMEM (Gibco, Carlsbad, CA, USA) containing 10% fetal bovine serum 6 h after transfection.

### 2.13. Statistical Analysis

The data is expressed as the mean ± SD. The differences among mean values were evaluated with a two-tailed Student’s *t*-test (for 2 groups) and analysis of variance (ANOVA; for >2 groups). All calculations and statistical tests were performed with SPSS (IBM SPSS statistics 26). For all analyses, *p* < 0.05 was considered significant.

## 3. Results

### 3.1. Inhibition of Tgf-β Enhanced DNA Damage and Decreased Cell Viability in 661W Cells

**Figure 1:**
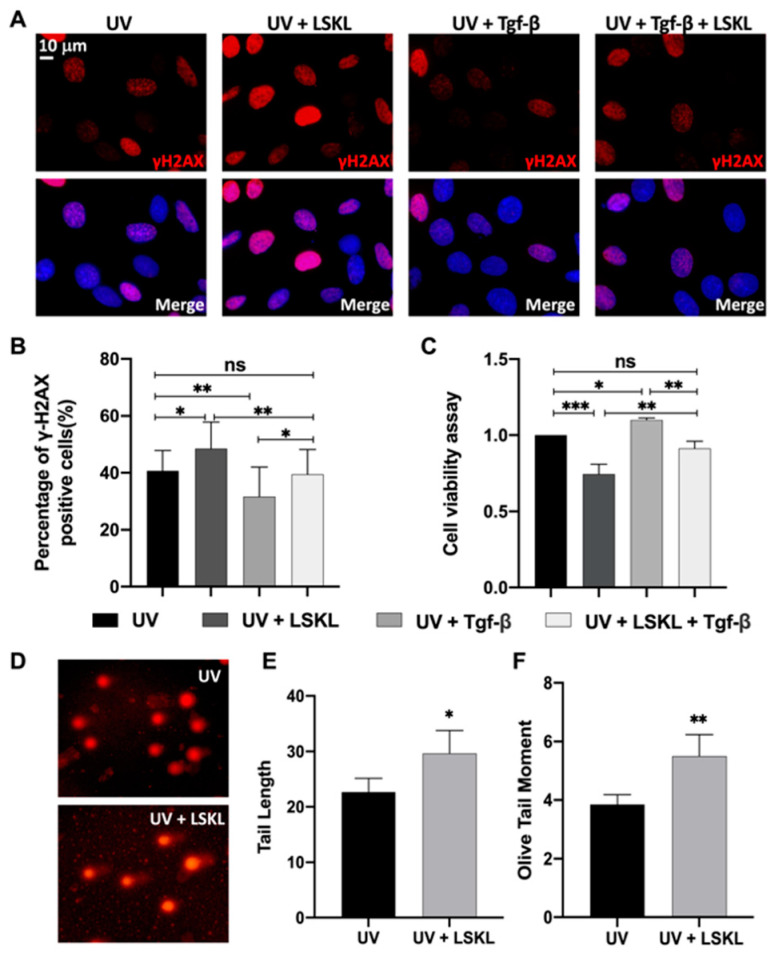
Tgf-β promoted DNA repair and cell viability in 661W cells. 661W cells were pretreated with LSKL (24 h), Tgf-β (1 h), both LSKL and Tgf-β or the control vehicle and exposed to UV irradiation. (**A**) The cells were then double-stained with γ-H2AX (red) and DAPI (blue). Scale bars: 10 μm. (**B**) The percentage of γ-H2AX-positive 661W cells was increased in the UV + LSKL group, decreased in the UV + Tgf-β group compared to the vehicle control and returned to the control level in the UV + LSKL + Tgf-β group. (**C**) CCK8 assay showed that the cell viability of 661W cells was markedly impaired by LSKL pretreatment, elevated by Tgf-β pretreatment and returned to control levels when treated with LSKL and Tgf-β together. (**D**) The alkaline comet assay was used to detect the level of DNA damage in 661W cells. The tail length (**E**) and the olive tail moment (**F**) were significantly upregulated by LSKL pretreatment. Data are presented as the mean ± SD from 3 independent experiments. * *p* < 0.05, ** p < 0.01, *** *p* < 0.001. Ns, no significance.

First, to verify the effect of Tgf-β and its inhibitor LSKL on DNA damage in the 661W cells, we measured the expression levels of γ-H2AX in the control (UV) and different experimental groups (LSKL + UV, Tgf-β + UV and LSKL + Tgf-β + UV) using immunofluorescence. Our data indicated that the level of γ-H2AX-positive cells is significantly downregulated after Tgf-β pretreatment (UV, 0.407 ± 0.060; Tgf-β + UV, 0.316 ± 0.116, ** *p* = 0.004), and it is significantly upregulated after LSKL pretreatment (UV, 0.407 ± 0.060; LSKL + UV, 0.753 ± 0.072, * *p* = 0.017). When pretreated with LSKL and Tgf-β together, the level of γ-H2AX-positive cells returned to the control level (UV, 0.407 ± 0.060; Tgf - β + LSKL + UV, 0.394 ± 0.054. *p* = 0.962) (Figure 1A,B). These results suggest that DNA damage induced by UV could be partially rescued by Tgf-β and aggravated by the Tgf-β inhibitor LSKL. In addition, the effect of LSKL on DNA damage was also measured in primary retinal neurons (Supplementary Figure S1A). The percentage of γ-H2AX-positive cells among MAP2-positive retinal neurons was significantly higher in LSKL-treated groups (UV, 0.19 ± 0.07; UV + LSKL, 0.26 ± 0.09, * *p* = 0.044, Supplementary Figure S1B). These results showed that the inhibition of Tgf-β could enhance DNA damage in both the 661W cells and primary retinal neurons. Accordingly, cell viability is significantly reduced upon LSKL pretreatment in the 661W cells (UV,1; LSKL + UV, 0.753 ± 0.072, *** *p* < 0.001), and it is significantly promoted upon Tgf-β pretreatment (UV, 1; Tgf-β + UV, 1.100 ± 0.015, * *p* = 0.044). When pretreated with LSKL and Tgf-β together, the level of cell viability returned to the control level (UV,1; Tgf-β + LSKL + UV, 0.923 ± 0.041, *p* = 0.075) (Figure 1C). The effect of LSKL on cell viability was also observed in primary retinal neurons (UV,1; LSKL + UV, 0.850 ± 0.092, * *p* = 0.047) (Supplementary Figure S1C).

To further confirm the effect of Tgf-β inhibition on DNA damage and repair in 661W cells, we performed an alkaline comet assay, which is an effective and reliable method for DNA damage detection in single cells. The 661W cells were pretreated with LSKL (10 μg/mL) or a control vehicle for 24 h before UV radiation (30 s). It was demonstrated by our previous study that a radiation dose of 30 J/m^2^ (254 nm) did not cause immediate cell death or apoptosis of the 661W cells while most cells showed apoptosis or death 12 h after radiation [6]. Therefore, the cells were harvested 30 min after UV radiation to ensure that the results of the comet assay represented the degree of DNA damage instead of the level of cell apoptosis. As shown in Figure 1D–F, the tail length (UV, 22.65 ± 2.53; UV + LSKL, 29.66 ± 4.14, * *p* = 0.017) and olive tail moment (UV, 3.85 ± 0.34; UV + LSKL, 5.50 ± 0.74, ** *p* = 0.006) of the comets were significantly increased in LSKL-treated groups. These results support that Tgf-β inhibition by LSKL could impair DNA stability in UV-radiated 661W cells.

### 3.2. Identification of DE-mRNAs and DE-lncRNAs between UV/Ctrl and UV/LSKL 661W Cells

**Figure 2:**
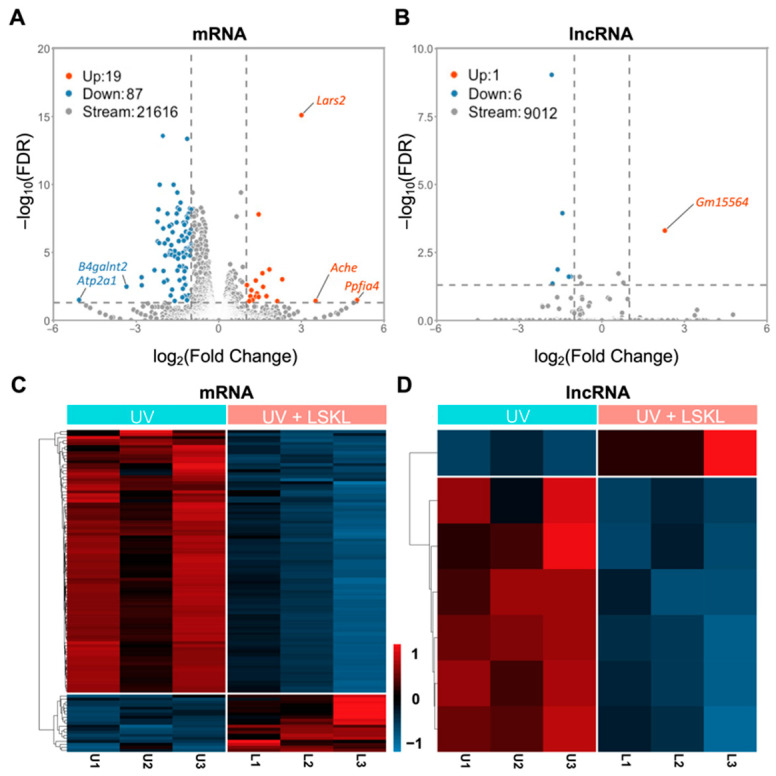
Identification of Tgf-β-associated DE-mRNAs and DE-lncRNAs in 661W cells. (**A**,**B**) Volcano plot illustrating the differential RNA expression level between UV/Ctrl and UV/LSKL 661W cells. The negative Log10 FDR adjusted *P* values (y-axis) are plotted against the log2 fold changes in expression (x-axis). (**C**,**D**) Heatmap illustrating the relative RNA expression of UV/Ctrl and UV/LSKL 661W cells. Microarray, *n* = 3 per group. DE-mRNAs: differentially expressed mRNAs; DE-lncRNAs: differentially expressed lncRNAs.

To explore the molecular mechanism underlying the protective effect of Tgf-β against DNA damage, we treated the 661W cells with LSKL before UV exposure and carried out transcriptome sequencing. A total of 53,379 RNA expression sequencing data were obtained, which were then successfully annotated with Ensemble’s annotation file, including 21,731 mRNAs and 9029 lncRNAs. A differential expression analysis was performed between the control (UV) and experimental (UV + LSKL) groups in the dataset and visualized as heatmaps and volcano plots (Figure 2). A total of 106 DE-mRNAs (19 upregulated and 87 downregulated) and 7 DE-lncRNAs (1 upregulated and 6 downregulated) were obtained with |log2FoldChange| > 1 and FDR < 0.05 (Table 1, Figure 2A,B). The gene category was further narrowed down by setting a more stringent threshold (|log2FoldChange| > 3 and FDR < 0.05), and five significant DE-mRNAs were obtained, including three upregulated (*Lars2*, *Ache*, *Ppfia4*) and two downregulated (*B4galnt2*, *Atp2a1*). Meanwhile, one upregulated DE-lncRNA (*Gm15564*) with a particularly high differential expression level was obtained with |log2FoldChange| > 2 and FDR < 0.05 (Figure 2B).

### 3.3. Enrichment Analysis and PPI Network Establishment Revealed Mechanisms and Pathways Associated with LSKL Prompting DNA Damage

**Figure 3:**
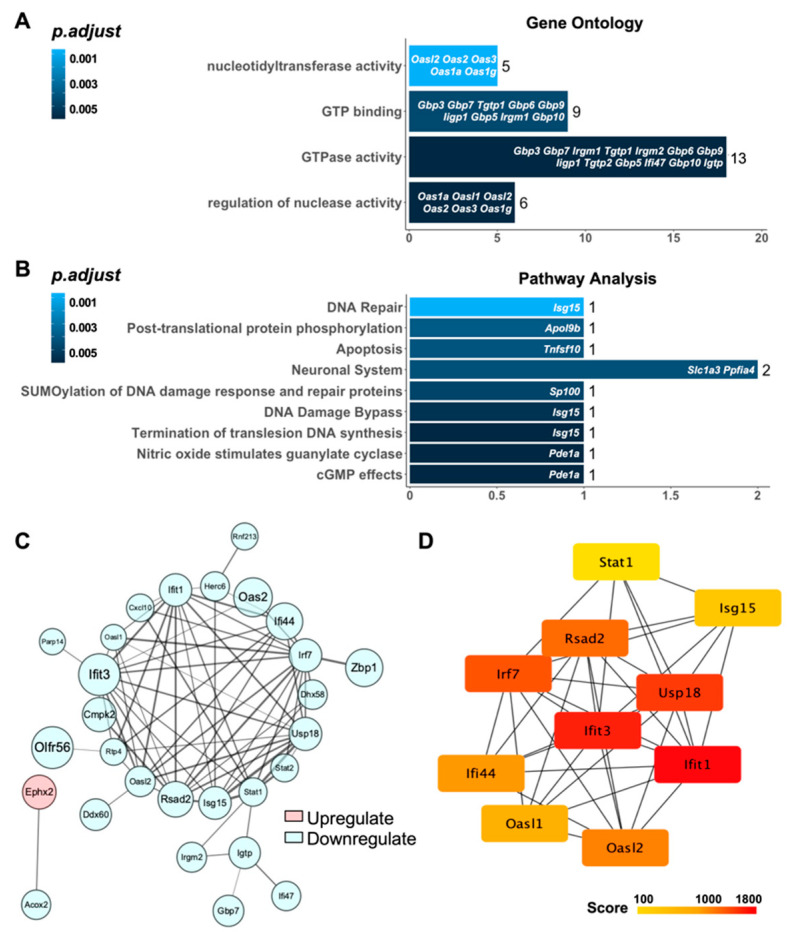
Pathway Analysis and PPI Network Establishment based on DE-mRNAs. (**A**) GO analysis revealed DE-mRNAs enriched in DNA damage- and repair-related GO terms. (**B**) The DE-mRNAs were significantly enriched in some DNA damage- and repair-related pathways in Reactome. (**C**) PPI network established based on DE-mRNAs. The node size was mapped according to |log2FoldChange| values, and the lines between the nodes were mapped by interaction scores. Pink represents the upregulated mRNA in the LSKL group compared to the UV control, and blue represents downregulated mRNAs. (**D**) The top 10 hub genes ranked according to the calculation of node connectivity by cytoHubba.

The DE-mRNAs were further analyzed with GO and pathway enrichment. When restricting to DNA damage and repair-related functions, the following GO terms were identified: nucleotidyl transferase activity, GTP binding, GTPase activity, and regulation of nuclease activity (Figure 3A). The DE-mRNAs were also significantly enriched in some DNA damage- and repair-related pathways in the Reactome, such as posttranslational protein phosphorylation, SUMOylation of DNA damage response and repair proteins, DNA damage bypass, termination of translesion DNA synthesis, nitric oxide-stimulated guanylate cyclase, and cGMP effects (Figure 3B). Taken together, these results showed that the DE-mRNAs we obtained are closely related to the DNA damage and repair process.

To further investigate the interactions among the proteins encoded by the identified DE-mRNAs, we conducted a PPI analysis. A PPI network with 28 nodes (27 downregulated and 1 upregulated) and 140 edges was obtained at the highest confidence level (0.9) (Figure 3C). The node size was mapped according to |log2FoldChange| values, and the lines between the nodes were mapped by interaction scores. As shown in Figure 3C, the lines between blue nodes are thicker, whereas the red nodes are only connected to one blue node. These results suggest that the proteins of downregulated genes have closer and more complex interactions with each other.

The PPI network was further analyzed to identify important central genes. According to the calculation of the node connectivity with cytoHubba, the top 10 genes were selected as hub genes (see Table 2) and visualized in Figure 3D. Our results show that all the central genes are downregulated genes. Among them, Isg15, Usp18, Oasl2, and Oasl1 were all enriched in terms related to DNA damage.

### 3.4. Establishment and Verification of a lncRNA-miRNA-mRNA ceRNA Network

**Figure 4:**
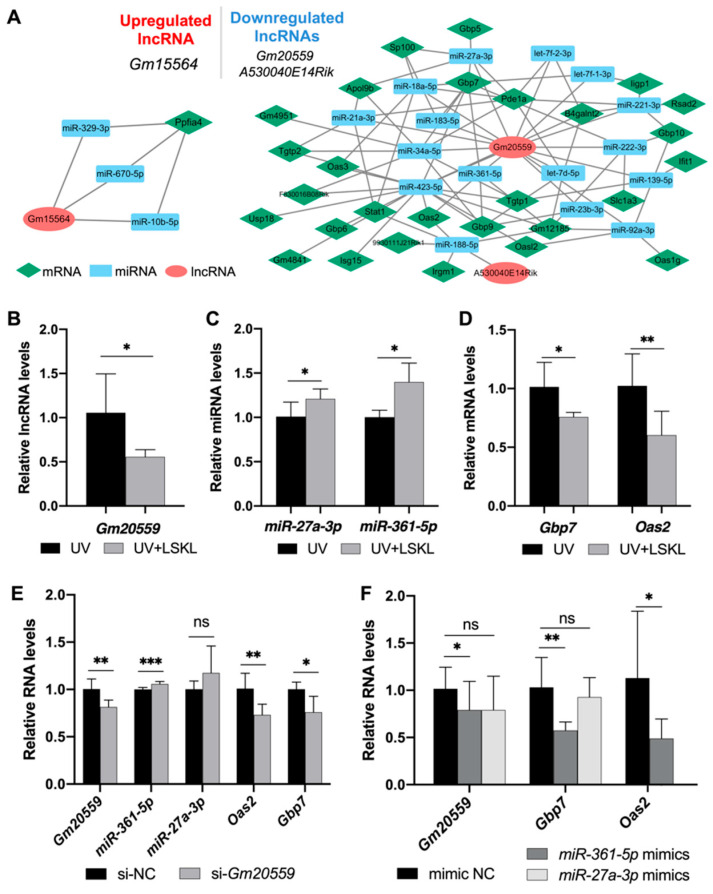
Construction and stepwise validation of a ceRNA network. (**A**) Up- and downregulated coexpression networks of DE-lncRNAs, DE-mRNAs and predicted target miRNAs. Red ovals, green triangles and blue rectangles represent lncRNAs, mRNAs, and miRNAs, respectively. The lines represent correlations. (**B**) RT-qPCR analysis of *Gm20559* in UV/Ctrl and UV/LSKL 661W cells. *Gm20559* was downregulated in UV/LSKL 661W cells compared with UV/ctrl. (**C**) *miR-27a-3p* and *miR-361-5p* were upregulated in UV/LSKL 661W cells compared to UV/ctrl. (**D**) *Oas2* and *Gbp7* were downregulated in UV/LSKL 661W cells compared to UV/ctrl. (**E**) 661W cells were transfected with si-*Gm20559* or si-NC for 48 h and exposed to UV radiation. The expression levels of *Gm20559*, *miR-361-5p*, *miR-27a-3p*, *Oas2* and *Gbp7* were then detected by RT-qPCR. (**F**) 661W cells were transfected with *miR-361-5p* mimics, *miR-27a-3p* or NC mimics for 48 h and exposed to UV radiation. Afterward, the expression levels of *Gm20559*, *Oas2* and *Gbp7* were detected by RT-qPCR. Data are presented as the mean ± SD from 3 independent experiments. * *p* < 0.05, ** *p <* 0.01, *** p < 0.001. Ns, no significance; RT-qPCR, reverse transcription-quantitative PCR.

To further explore the potential noncoding regulatory mechanisms underlying LSKL-mediated DNA damage, we established a ceRNA network through in silico prediction and experimental validation. First, we used an online database to predict the target miRNAs of the hub genes, the DE-mRNAs enriched in DNA damage/repair-related GO terms, and all DE-lncRNAs. The predicted results were processed in R, including intersection and filtration, and a final ceRNA network containing 3 lncRNAs, 19 miRNAs, and 29 mRNAs was obtained. We then separated the ceRNA network into up- and downregulated subgroups based on the expression profiles of DE-lncRNAs. The upregulated ceRNA network (up_ceRNA network) consisted of 2 lncRNAs (*Gm20559* and *A530040E14Rik*), 16 miRNAs, and 28 mRNAs while the downregulated ceRNA network (down_ceRNA network) consisted of 1 lncRNA (*Gm15564*), 3 miRNAs, and 1 mRNA (Figure 4A).

The predicted ceRNA network was further validated in the 661W cells with RT-qPCR. Consistent with our prediction, *Gm20559* was downregulated after LSKL treatment (UV, 1.06 ± 0.44; LSKL + UV, 0.57 ± 0.10, * *p* = 0.0497) (Figure 4B, Supplementary Figure S2A). Moreover, among the miRNAs corresponding to *Gm20559*, *miR-27a-3p* (UV, 1.01 ± 0.16; LSKL + UV, 1.21 ± 0.11, * *p* = 0.022) and *miR-361-5p* (UV, 1.00 ± 0.08; LSKL + UV, 1.40 ± 0.21, * *p* = 0.018) were upregulated after LSKL treatment (Figure 4C, Supplementary Figure S2C). Furthermore, among the 8 target genes of *miR-27a-3p* and *miR-361-5p*, *Gbp7* (UV, 1.01 ± 0.20; LSKL + UV, 0.75 ± 0.04, * *p* = 0.023) and *Oas2* (UV, 1.02 ± 0.27; LSKL + UV, 0.60 ± 0.20, ** *p* = 0.003) were downregulated after LSKL treatment (Figure 4D, Supplementary Figure S2B). Taken together, the results showed that 3 lncRNA-miRNA-mRNA axes (*Gm20559*/*miR-27a-3p*/*Gbp7*, *Gm20559*/*miR-361-5p*/*Gbp7*, and *Gm20559*/*miR-361-5p*/*Oas2*) might be involved in LSKL-mediated DNA damage progression.

To further validate the mutual influences between lncRNAs, miRNAs and mRNAs, dynamic changes in miRNAs and mRNAs were detected upon knockdown of *Gm20559*. We utilized 2 siRNAs (si*Gm20559*#1 and si*Gm20559*#2) to knockdown *Gm20559* expression (si-NC + UV, 1.00 ± 0.11; si-*Gm20559* + UV, 0.81 ± 0.07, * *p* = 0.011) in 661W cells. As a result, *miR-361-5p* (si-NC + UV, 1.00 ± 0.02; si-*Gm20559* + UV, 1.05 ± 0.03, *** *p <* 0.001) was upregulated, and *Oas2* (si-NC + UV, 1.01 ± 0.16; si-*Gm20559* + UV, 0.75 ± 0.17, ** *p* = 0.010) and *Gbp7* (si-NC + UV, 1.00 ± 0.08; si-*Gm20559* + UV, 0.76 ± 0.17, * *p* = 0.017) were downregulated in *Gm20559* knockdown cells, while *miR-27a-3p* expression was not significantly altered (si-NC + UV, 1.00 ± 0.09; si-*Gm20559* + UV, 1.17 ± 0.28, *p* = 0.388) (Figure 4E). Moreover, to further validate the interactions between miRNAs and their target mRNAs, we transfected the 661W cells with *miR-361-5p* and *miR-27a-3p* mimics. As shown in Figure 4F, *Oas2* (mimics NC + UV, 1.13 ± 0.70; *miR-361-5p* mimics + UV, 0.49 ± 0.21, * *p* = 0.013) and *Gbp7* (mimics NC + UV, 1.03 ± 0.32; *miR-361-5p* mimics + UV, 0.57 ± 0.09, ** *p* = 0.009) were downregulated in cells treated with *miR-361-5p* mimics. Moreover, *Gm20559* was also downregulated in the *miR-361-5p* mimic group (mimics NC + UV, 1.02 ± 0.23, *miR-361-5p* mimics + UV, 0.79 ± 0.30, * *p* = 0.024, Figure 4F). However, there were no significant differences in *Gbp7* (mimics NC + UV, 1.03 ± 0.32; *miR-27a-3p* mimics + UV, 0.93 ± 0.21, *p* = 0.719) and *Gm20559* expression (mimics NC + UV, 1.02 ± 0.23; *miR-27a-3p* mimics + UV, 0.79 ± 0.36, *p* = 0.116) between the *miR-27a-3p* mimic and control groups (Figure 4F). Taken together, the results suggested ceRNA-regulatory mechanisms among *Gm20559*-*miR-361-5p*-*Gbp7*/*Oas2*, which might serve important roles in DNA damage and repair downstream from Tgf-β signaling.

We further validated the expression profiles of the ceRNA components (*Gm20559*-*miR-361-5p*-*Gbp7*/*Oas2*) after LSKL treatment in primary retinal neurons. As shown in Supplementary Figure S3A–C, *Gm20559* (UV, 1.11 ± 0.65; LSKL + UV, 0.92 ± 0.53, *** *p* = 0.0002), *Gbp7* (UV, 1.03 ± 0.32; LSKL + UV, 0.70 ± 0.13, * *p* = 0.022), and *Oas2* (UV, 1.05 ± 0.37; LSKL + UV, 0.80 ± 0.54, *p* = 0.41) were downregulated in LSKL-treated primary retinal neurons, while *miR-361-5p* was upregulated (UV, 1.00 ± 0.08; LSKL + UV, 1.29 ± 0.10, * *p* = 0.033). As such, similar expression profiles of these ceRNA components were identified in primary retinal neurons, supporting the general nature of this downstream ceRNA-regulatory mechanism of Tgf-β.

### 3.5. Functional Analysis of the ceRNA Network

**Figure 5:**
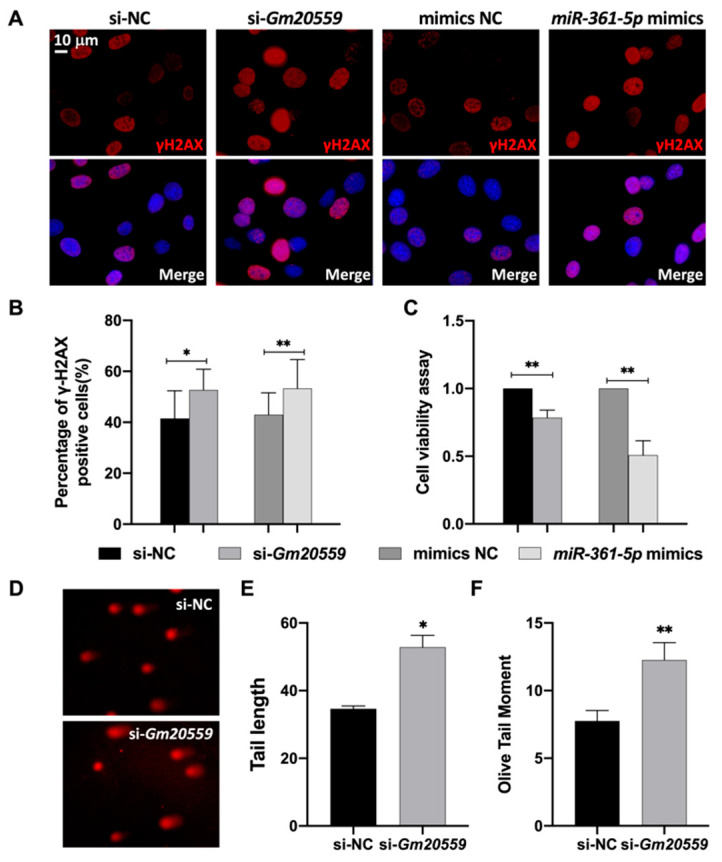
*Gm20559* knockdown or *miR-361-5p* mimics enhanced DNA damage and decreased cell viability in 661W cells. 661W cells were transfected with si-NC, si-*Gm20559*, NC mimics, or *miR-361-5p* mimics and exposed to UV irradiation. (**A**) The cells were then double-stained with γ-H2AX (red) and DAPI (blue). Scale bars: 10 μm. (**B**) The percentage of γ-H2AX-positive 661W cells was increased in si-*Gm20559* cells compared with si-NC and was increased in cells treated with *miR-361-5p* mimics compared with NC mimics. (**C**) CCK8 assay showed that the cell viability of 661W cells was markedly impaired by *Gm20559* knockdown or *miR-361-5p* mimics. (**D**) The alkaline comet assay was used to detect the level of DNA damage in 661W cells. The tail length (**E**) and the olive tail moment (**F**) were significantly upregulated in *Gm20559* knockdown cells. Data are presented as the mean ± SD from 3 independent experiments. * *p* < 0.05, ** *p* < 0.01.

To further confirm the function of the components in the ceRNA axes with respect to DNA damage and repair, we transfected the 661W cells with si-*Gm20559*, *miR-361-5p* mimics, and controls. At 48 h after transfection, the level of γ-H2AX was detected in the 661W cells with immunofluorescence. As shown in Figure 5A,B, knockdown of *Gm20559* significantly enhanced UV-induced γ-H2AX expression (si-NC + UV, 0.41 ± 0.02; si-*Gm20559* + UV, 0.53 ± 0.04; * *p* = 0.015). The *miR-361-5p* mimics also significantly enhanced γ-H2AX expression (mimics NC + UV, 0.43 ± 0.03; *miR-361-5p* mimics + UV, 0.53 ± 0.02; ** *p* = 0.004). Moreover, cell viability was impaired by *Gm20559* knockdown (si-NC + UV, 1; si-*Gm20559* + UV, 0.785 ± 0.055; ** *p* = 0.003), and *miR-361-5p* mimics also impaired cell viability mimics NC + UV, 1; *miR-361-5p* mimics + UV, 0.508 ± 0.107; ** *p* = 0.001) (Figure 5C). Taken together, these results suggested that the downregulation of *Gm20559* and the upregulation of *miR-361-5p* in the LSKL-mediated ceRNA network could enhance DNA damage and impair cell viability in 661W cells.

To further confirm the effect of *Gm20559* knockdown on DNA damage in 661W cells, we carried out an alkaline comet assay on si-NC and si-*Gm20559* groups (Figure 5D). As shown in Figure 5E,F, *Gm20559* knockdown significantly upregulated the tail length (si-NC + UV, 34.61 ± 0.88; si-*Gm20559* + UV, 52.81 ± 3.54; * *p* = 0.017) and olive tail moment (si-NC + UV, 7.76 ± 0.77; si-*Gm20559* + UV, 12.26 ± 1.28; ** *p* = 0.006) of the comets. These results support that *Gm20559* could further impair DNA stability in UV-radiated 661W cells.

## 4. Discussion

In the present study, we found that Tgf-β attenuated DNA damage in PRs upon UV radiation while its inhibitor LSKL promoted DNA damage. Moreover, multiple downstream DNA damage/repair-related mRNAs and pathways of Tgf-β signaling were identified through transcriptome sequencing and bioinformatics analysis. Furthermore, 2 downstream lncRNA-miRNA-mRNA axes (*Gm20559*-*miR-361-5p*-*Gbp7*/*Oas2*) of Tgf-β signaling were demonstrated through the ceRNA network prediction followed by stepwise experimental verification, which revealed the noncoding mechanisms underlying the Tgf-β-regulated DNA damage and repair process in 661W cells. Taken together, our study confirmed the protective role of Tgf-β against DNA damage in PRs and further revealed its underlying mechanisms.

In our study, we first demonstrated that Tgf-β could attenuate DNA damage and improve cell viability of UV-radiated 661W cells (Figure 1A–F), which is supported by previous studies [11,12,15,32,33,34]. The general neuroprotective role of Tgf-β against various harmful stimuli has been demonstrated [35,36,37,38,39]. In terms of the role of Tgf-β and photoreceptor damage in ocular diseases, a recent study showed that Tgf-β signaling is vital for photoreceptor viability, while deletion of Tgf-β signaling resulted in thinner outer nuclear layer and accelerated retinal degeneration [38]. More importantly, Tgf-β exerts its neuroprotective role through direct impact on photoreceptors [35], which is also consistent with our study. The capability of maintaining DNA stability could partially accounts for the neuroprotective role of Tgf-β. For example, Tgf-β could induce Gadd45b expression, enhance DNA repair, and therefore protect retinal ganglion cells [11]. Moreover, we previously found that Tgf-β1 was significantly downregulated upon UV treatment in the 661W cells, and the inhibition of Tgf-β1 enhanced UV-induced DNA damage [6]. Although studies showed that Tgf-β could protect neurons through maintaining DNA stability, some studies have reached different conclusions. For example, Hicks et al. reported that Tgf-β potentiated ethanol-induced DNA damage in neural stem cells [32]. Moreover, Tgf-β pathway inhibition promotes DNA repair and improves hematopoietic stem cell (HSC) survival in Fanconi anemia [40]. Notably, inhibition of Tgf-β signaling results in elevated homologous recombination (HR) repair with a concomitant decrease in nonhomologous end-joining (NHEJ). As such, we speculate that differences in cell types (proliferative/nonproliferative) and forms of DNA damage might account for the discrepancies between different studies.

Moreover, the inhibition of Tgf-β signaling affected a variety of biological functions and pathways associated with DNA damage and repair, such as nucleotidyltransferase activity, regulation of nuclease activity, posttranscriptional modification of DNA damage response, and repair proteins and translesion DNA synthesis (TLS) (Figure 3A,B). Among these, nucleotidyltransferases are a family of enzymes that can add nucleotides to substrates such as DNA, and they are involved in DNA damage and repair [41]. For example, Tang et al. reported that DNA polymerase β (Pol β) could act as a nucleotidyltransferase and is involved in the repair pathway for single nucleotide base excision repair (BER) [42]. Second, nucleases are various enzymes that promote the hydrolysis of nucleic acids; these activities are key for most DNA repair systems, including BER, NHEJ, and HR [43]. Last, TLS is a DNA damage tolerance mechanism allowing cells to bypass DNA lesions, thus avoiding the collapse of replication forks [44]. Changes in these pathways further confirmed that Tgf-β is involved in DNA repair in PRs.

Furthermore, we identified novel and important downstream molecular targets of Tgf-β signaling effectively through the combination of RNA-seq and bioinformatic analysis, which is one of the strengths of this study. *Isg15*, *Usp18*, *Oasl2*, and *Oasl1* were identified as hub genes enriched in terms related to DNA damage and repair, which were all downregulated upon LSKL treatment (Figure 3C,D). Isg15 is a ubiquitin-like protein (UBL) which has been recognized as an important regulator of genome stability through covalent modification of—or noncovalent interaction with—key proteins involved in the DNA damage response (DDR) [44]. Associated pathways include p53 signaling, replication fork acceleration, and TLS. *Usp18* is also a key modulator of TLS. It acts as an Isg15-specific protease responsible for the removal of Isg15 from substrates and is responsible for resumption of DNA replication [45]. Downregulation of these two genes implies reduced TLS capability, resulting in unsuccessful DNA repair. Moreover, *Oasl1* and *Oasl2* are associated with the regulation of nuclease activity. Accordingly, they have 2′-5′ oligoadenylate synthase (OAS)-like qualities, such as activation by interferon (IFN) and binding to dsRNA [46]. However, few studies have reported the relationship between Oasl and DNA damage and repair, which requires further study.

To comprehensively depict the interactions of both protein-coding and noncoding RNAs, we then established a ceRNA network through prediction and stepwise experimental validation (Figure 4A–D). Several novel molecular targets associated with retinal DNA damage and their interactions were identified based on the ceRNA network. The combination of bioinformatic analysis and experimental validation improved the reliability of these results. We found that *Gm20559* knockdown caused the release and upregulation of *miR-361-5p*, which then reduced the expression of its target mRNAs *Oas2* and *Gbp7* (Figure 4E,F). Most importantly, our study indicated that *Gm20559*/*miR-361-5p* could stabilize/impair DNA stability in PRs, respectively (Figure 5A–F), which is indirectly supported by previous studies. For example, it was demonstrated that *miR-361-5p* was associated with PARP1, ubiquitin protein ligase E3 component N-recognin 5 (UBR5) and ataxia-telangiectasia mutated interactor (ATMIN) in tumors, which are enriched in DNA damage and repair [47,48]. Of note is the fact that *miR-361-5p* upregulation could reduce UBR5 expression [47], therefore inhibiting ATMIN ubiquitination, attenuating the restoration of ATM and impairing DNA repair [49]. Among the other ceRNA components, *Gm20559* is a lncRNA with 3 exons located on chromosome 6. Limited studies have shown that *Gm20559* is associated with neuronal diseases, such as Alzheimer’s Disease [50] and rabies virus infection of the mouse brain [51], while the biofunction of *Gm20559* is largely unknown. This is the first study demonstrating that *Gm20559* could regulate DNA repair in retinal 661W cells. Moreover, Gbp7 belongs to the guanylate-binding protein family, which is part of an IFN-γ-inducible guanosine triphosphatase (GTPase) superfamily [52]. GTPases are actively involved in various DNA repair processes [53,54], which might account for the regulatory role of *Gbp7* in DNA repair. Similar to *Gbp7*, *Oas2* could also be induced by IFN and serve a role in host defense against infection [55]. Previous studies supported the assertion that the upregulation of *Oas2* could enhance resistance to DNA damage and improve cell viability in cancers [56,57]. However, few studies have discussed the function of *Oas2* in neuronal pathologies. As such, further studies are required to explore the roles of *Gm20559*, *Gbp7*, and *Oas2* in DNA damage and repair.

Of note is that inhibition of Tgf-β signaling also accelerated UV-induced DNA damage in primary retinal neurons, and similar expression profiles of *Gm20559* and *miR-361-5p* were also identified. In addition, the general neuroprotective role of Tgf-β was supported by multiple studies as mentioned above. A great portion of downstream hub genes and ceRNA components of Tgf-β signaling were also shown to modulate DNA damage and repair in other tissues. Taken together, these findings and studies supported the general nature of our results. However, there are some limitations in this study. For example, this study was only performed in cell culture, and some experiments were performed on only one cell line. Thus, further studies are required to validate the effects of Tgf-β and its downstream ceRNA network on DNA damage in animal models and the functions of the ceRNA components in other cell lines.

In conclusion, our study demonstrated a protective role of Tgf-β signaling upon DNA damage in PRs. Tgf-β signaling affected various DNA damage/repair-related biological functions and pathways. Moreover, the comprehensive posttranscriptional regulatory network formed by ceRNA activity could greatly broaden the understanding of Tgf-β mediated DNA stability, provide novel biomarkers for DNA damage and repair in PRs, and indicate treatment targets for relevant diseases.

## Figures and Tables

**Table 1:** The differentially expressed mRNAs and lncRNAs between two UV irradiated 661W cells with or without LSKL preprocessing.

	DEmRNAs	DElncRNAs
Upregulated	(Count: 19) *Lars2*, *Prl2c3*, *Rcan2*, *Ephx2*, *Tafa5*, *Sbsn*, *Tnnt2*, *Tagln*, *Id2*, *Sh3tc1*, *Sh3d21*, *Inpp4b*, *Actg2*, *Dnase1l2*, *Inka2*, *Ppfia4*, *Ache*, *Slurp1*, *Atoh8*	(Count: 1) *Gm15564*
Downregulated	(Count: 87) *Cxcl10*, *Olfr56*, *Gbp3*, *Ifit3*, *Isg15*, *Gbp7*, *Ifit1*, *Irgm1*, *Samd9l*, *Tgtp1*, *Igtp*, *Gbp6*, *Usp18*, *Gask1b*, *Rnf213*, *Xaf1*, *Trim12c*, *Ifit3b*, *Irf7*, *Trim34a*, *Oas1a*, *Herc6*, *Postn*, *Ifi44*, *Gvin1*, *Oasl1*, *Stat1*, *Phf11d*, *Gbp9*, *Sp100*, *Oasl2*, *Stat2*, *Zbp1*, *Gm4841*, *Ifit1bl2*, *Tnfsf10*, *9930111J21Rik1*, *Iigp1*, *Trim30d*, *Slfn2*, *Apol9b*, *Trim12a*, *Trim30a*, *Rtp4*, *H2-T23*, *Avil*, *Cmpk2*, *H2-T24*, *Ifi27l2a*, *Irgm2*, *Gm4951*, *Phf11b*, *Gm4070*, *Rsad2*, *F830016B08Rik*, *Ifi203*, *Oas2*, *Parp14*, *Tgtp2*, *Dhx58*, *Acox2*, *Gbp5*, *Slfn8*, *Ddx60*, *Sp110*, *Misp*, *Oas3*, *Mpeg1*, *Oas1g*, *Batf2*, *Ifi47*, *Slc1a3*, *Ranbp3l*, *Perm1*, *Car6*, *Gbp4*, *Pde1a*, *Ly6f*, *B4galnt2*, *AA467197*, *Gm12185*, *Rgcc*, *Klf15*, *Zfp781*, *Sema3d*, *Atp2a1*, *Gbp10*	(Count: 6) *Gm2619*, *Gm45418*, *AW011738*, *Gm9801*, *Gm20559*, *A530040E14Rik*

DEmRNAs/DElncRNAs were set as adjust *p*-value < 0.05 and |log2FC| > 1; DEmRNAs: differentially expressed mRNAs; DElncRNAs: differentially expressed lncRNAs.

**Table 2:** Rank of hub genes (top 10).

Rank	Name	Score
1	*Ifit1*	1810
2	*Ifit3*	1809
3	*Usp18*	1806
4	*Irf7*	1705
5	*Rsad2*	1568
6	*Oasl2*	1561
7	*Ifi44*	848
8	*Oasl1*	720
9	*Isg15*	248
10	*Stat1*	134

## Data Availability

Data is contained within the paper and the Supplementary Material.

## Supplementary Materials

The following are available online at https://www.mdpi.com/article/10.3390/genes13112140/s1, Supplementary Figure S1: Tgf-β inhibition impaired DNA repair and cell viability in primary retinal neurons.; Supplementary Figure S2: Relative RNA levels of lncRNA, miRNA, and mRNA components in the ceRNA network after LSKL treatment in 661W cells.; Supplementary Figure S3: Relative RNA levels of lncRNA, miRNA, and mRNA components in the ceRNA network after LSKL treatment in primary retinal neurons.; Supplementary Table S1: Primers used for RT-qPCR.; Supplementary Table S2: Sequences of siRNAs and miRNA mimics.; Supplementary Table S3: Read counts from RNA-sequencing.

## References

[1] Gallenga C.E., Lonardi M., Pacetti S., Violanti S.S., Tassinari P., Di Virgilio F., Tognon M., Perri P. (2021). Molecular Mechanisms Related to Oxidative Stress in Retinitis Pigmentosa. Antioxidants.

[2] Liao C., Cai B., Feng Y., Chen J., Wu Y., Zhuang J., Liu Z., Wu Y. (2020). Activation of JNK signaling promotes all-trans-retinal-induced photoreceptor apoptosis in mice. J. Biol. Chem..

[3] Toma C., De Cillà S., Palumbo A., Garhwal D.P., Grossini E. (2021). Oxidative and Nitrosative Stress in Age-Related Macular Degeneration: A Review of Their Role in Different Stages of Disease. Antioxidants.

[4] Szaflik J.P., Janik-Papis K., Synowiec E., Ksiazek D., Zaras M., Wozniak K., Szaflik J., Blasiak J. (2009). DNA damage and repair in age-related macular degeneration. Mutat. Res..

[5] Kaur J., Mencl S., Sahaboglu A., Farinelli P., van Veen T., Zrenner E., Ekström P., Paquet-Durand F., Arango-Gonzalez B. (2011). Calpain and PARP activation during photoreceptor cell death in P23H and S334ter rhodopsin mutant rats. PLoS ONE.

[6] Chen P., Liu C., Zhang J., Chen X., Liu X., He S., He A., Chen S., Qiu J., Li Y. (2022). Tsp-1 is involved in DNA stability through Tgf-β1 activation domain in cone photoreceptor 661 W cells. Cell Tissue Res..

[7] Hegde M.L., Bohr V.A., Mitra S. (2017). DNA damage responses in central nervous system and age-associated neurodegeneration. Mech. Ageing Dev..

[8] Kinner A., Wu W., Staudt C., Iliakis G. (2008). Gamma-H2AX in recognition and signaling of DNA double-strand breaks in the context of chromatin. Nucleic Acids Res..

[9] Yang Y., Wu N., Tian S., Li F., Hu H., Chen P., Cai X., Xu L., Zhang J., Chen Z. (2016). Lithium promotes DNA stability and survival of ischemic retinal neurocytes by upregulating DNA ligase IV. Cell Death Dis..

[10] Liu Q., Lopez K., Murnane J., Humphrey T., Barcellos-Hoff M.H. (2019). Misrepair in Context: TGFβ Regulation of DNA Repair. Front. Oncol..

[11] Liu B., Sun X., Suyeoka G., Garcia J.G., Leiderman Y.I. (2013). TGFβ signaling induces expression of Gadd45b in retinal ganglion cells. Investig. Ophthalmol. Vis. Sci..

[12] Li Y., Liu Y., Chiang Y.J., Huang F., Li Y., Li X., Ning Y., Zhang W., Deng H., Chen Y.G. (2019). DNA Damage Activates TGF-β Signaling via ATM-c-Cbl-Mediated Stabilization of the Type II Receptor TβRII. Cell Rep..

[13] Zi Z. (2019). Molecular Engineering of the TGF-β Signaling Pathway. J. Mol. Biol..

[14] Hata A., Chen Y.G. (2016). TGF-β Signaling from Receptors to Smads. Cold Spring Harb. Perspect. Biol..

[15] An Y.S., Kim M.R., Lee S.S., Lee Y.S., Chung E., Song J.Y., Lee J., Yi J.Y. (2013). TGF-β signaling plays an important role in resisting γ-irradiation. Exp. Cell Res..

[16] Liang J., Liao J., Liu T., Wang Y., Wen J., Cai N., Huang Z., Xu W., Li G., Ding Z. (2020). Comprehensive analysis of TGF-β-induced mRNAs and ncRNAs in hepatocellular carcinoma. Aging.

[17] Lai X.N., Li J., Tang L.B., Chen W.T., Zhang L., Xiong L.X. (2020). MiRNAs and LncRNAs: Dual Roles in TGF-β Signaling-Regulated Metastasis in Lung Cancer. Int. J. Mol. Sci..

[18] Salmena L., Poliseno L., Tay Y., Kats L., Pandolfi P.P. (2011). A ceRNA hypothesis: The Rosetta Stone of a hidden RNA language?. Cell.

[19] Zheng H., Jarvis I.W.H., Bottai M., Dreij K., Stenius U. (2019). TGF beta promotes repair of bulky DNA damage through increased ERCC1/XPF and ERCC1/XPA interaction. Carcinogenesis.

[20] Durinck S., Moreau Y., Kasprzyk A., Davis S., De Moor B., Brazma A., Huber W. (2005). BioMart and Bioconductor: A powerful link between biological databases and microarray data analysis. Bioinformatics.

[21] Durinck S., Spellman P.T., Birney E., Huber W. (2009). Mapping identifiers for the integration of genomic datasets with the R/Bioconductor package biomaRt. Nat. Protoc..

[22] McCarthy D.J., Chen Y., Smyth G.K. (2012). Differential expression analysis of multifactor RNA-Seq experiments with respect to biological variation. Nucleic Acids Res..

[23] Robinson M.D., McCarthy D.J., Smyth G.K. (2010). edgeR: A Bioconductor package for differential expression analysis of digital gene expression data. Bioinformatics.

[24] Ginestet C. (2011). ggplot2: Elegant Graphics for Data Analysis. J. R. Stat. Soc. Ser. a-Stat. Soc..

[25] Yu G., Wang L.-G., Han Y., He Q.-Y. (2012). clusterProfiler: An R Package for Comparing Biological Themes Among Gene Clusters. Omics-A J. Integr. Biol..

[26] Szklarczyk D., Franceschini A., Kuhn M., Simonovic M., Roth A., Minguez P., Doerks T., Stark M., Muller J., Bork P. (2011). The STRING database in 2011: Functional interaction networks of proteins, globally integrated and scored. Nucleic Acids Res..

[27] Shannon P., Markiel A., Ozier O., Baliga N.S., Wang J.T., Ramage D., Amin N., Schwikowski B., Ideker T. (2003). Cytoscape: A software environment for integrated models of biomolecular interaction networks. Genome Res..

[28] Wang W., Lou W., Ding B., Yang B., Lu H., Kong Q., Fan W. (2019). A novel mRNA-miRNA-lncRNA competing endogenous RNA triple sub-network associated with prognosis of pancreatic cancer. Aging-Us.

[29] Karagkouni D., Paraskevopoulou M.D., Tastsoglou S., Skoufos G., Karavangeli A., Pierros V., Zacharopoulou E., Hatzigeorgiou A.G. (2020). DIANA-LncBase v3: Indexing experimentally supported miRNA targets on non-coding transcripts. Nucleic Acids Res..

[30] Sticht C., De La Torre C., Parveen A., Gretz N. (2018). miRWalk: An online resource for prediction of microRNA binding sites. PLoS ONE.

[31] Smoot M.E., Ono K., Ruscheinski J., Wang P.L., Ideker T. (2011). Cytoscape 2.8: New featuRes. for data integration and network visualization. Bioinformatics.

[32] Hicks S.D., Miller M.W. (2019). Ethanol-induced DNA repair in neural stem cells is transforming growth factor β1-dependent. Exp. Neurol..

[33] Lee J., Kim M.R., Kim H.J., An Y.S., Yi J.Y. (2016). TGF-β1 accelerates the DNA damage response in epithelial cells via Smad signaling. BioChem. Biophys. Res. Commun..

[34] Liu Q., Palomero L., Moore J., Guix I., Espín R., Aytés A., Mao J.H., Paulovich A.G., Whiteaker J.R., Ivey R.G. (2021). Loss of TGFβ signaling increases alternative end-joining DNA repair that sensitizes to genotoxic therapies across cancer types. Sci. Transl. Med..

[35] Braunger B.M., Pielmeier S., Demmer C., Landstorfer V., Kawall D., Abramov N., Leibinger M., Kleiter I., Fischer D., Jägle H. (2013). TGF-β signaling protects retinal neurons from programmed cell death during the development of the mammalian eye. J. Neurosci..

[36] Henrich-Noack P., Prehn J.H., Krieglstein J. (1996). TGF-beta 1 protects hippocampal neurons against degeneration caused by transient global ischemia. Dose-response relationship and potential neuroprotective mechanisms. Stroke.

[37] Tesseur I., Nguyen A., Chang B., Li L., Woodling N.S., Wyss-Coray T., Luo J. (2017). Deficiency in Neuronal TGF-β Signaling Leads to Nigrostriatal Degeneration and Activation of TGF-β Signaling Protects against MPTP Neurotoxicity in Mice. J. Neurosci..

[38] Bielmeier C.B., Schmitt S.I., Kleefeldt N., Boneva S.K., Schlecht A., Vallon M., Tamm E.R., Hillenkamp J., Ergün S., Neueder A. (2022). Deficiency in Retinal TGFβ Signaling Aggravates Neurodegeneration by Modulating Pro-Apoptotic and MAP Kinase Pathways. Int. J. Mol. Sci..

[39] Conedera F.M., Quintela Pousa A.M., Presby D.M., Mercader N., Enzmann V., Tschopp M. (2021). Diverse Signaling by TGFβ Isoforms in Response to Focal Injury is Associated with Either Retinal Regeneration or Reactive Gliosis. Cell Mol. NeuroBiol..

[40] Zhang H., Kozono D.E., O’Connor K.W., Vidal-Cardenas S., Rousseau A., Hamilton A., Moreau L., Gaudiano E.F., Greenberger J., Bagby G. (2016). TGF-β Inhibition Rescues Hematopoietic Stem Cell Defects and Bone Marrow Failure in Fanconi Anemia. Cell Stem Cell.

[41] Ranjitkar S., Duan J.E., Srirattana K., Alqahtani F., Tulman E.R., Mandoiu I., Venkitanarayanan K., Tian X. (2022). Transcriptomic Responses of Mycoplasma bovis Upon Treatments of trans-Cinnamaldehyde, Carvacrol, and Eugenol. Front. MicroBiol..

[42] Tang K.H., Niebuhr M., Aulabaugh A., Tsai M.D. (2008). Solution structuRes. of 2:1 and 1:1 DNA polymerase-DNA complexes probed by ultracentrifugation and small-angle X-ray scattering. Nucleic Acids Res..

[43] Miyazono K.I., Ishino S., Makita N., Ito T., Ishino Y., Tanokura M. (2018). Crystal structure of the novel lesion-specific endonuclease PfuEndoQ from *Pyrococcus furiosus*. Nucleic Acids Res..

[44] Sandy Z., da Costa I.C., Schmidt C.K. (2020). More than Meets the ISG15: Emerging Roles in the DNA Damage Response and Beyond. Biomolecules.

[45] Malakhov M.P., Malakhova O.A., Kim K.I., Ritchie K.J., Zhang D.E. (2002). UBP43 (USP18) specifically removes ISG15 from conjugated proteins. J. Biol. Chem..

[46] Choi U.Y., Kang J.S., Hwang Y.S., Kim Y.J. (2015). Oligoadenylate synthase-like (OASL) proteins: Dual functions and associations with diseases. Exp. Mol. Med..

[47] Jia J., Ouyang Z., Wang M., Ma W., Liu M., Zhang M., Yu M. (2021). MicroRNA-361-5p slows down gliomas development through regulating UBR5 to elevate ATMIN protein expression. Cell Death Dis..

[48] Tommasi S., Pinto R., Danza K., Pilato B., Palumbo O., Micale L., De Summa S. (2016). miR-151-5p, targeting chromatin remodeler SMARCA5, as a marker for the BRCAness phenotype. Oncotarget.

[49] Li C.G., Mahon C., Sweeney N.M., Verschueren E., Kantamani V., Li D., Hennigs J.K., Marciano D.P., Diebold I., Abu-Halawa O. (2019). PPARγ Interaction with UBR5/ATMIN Promotes DNA Repair to Maintain Endothelial Homeostasis. Cell Rep..

[50] Wan G., Zhou W., Hu Y., Ma R., Jin S., Liu G., Jiang Q. (2016). Transcriptional Regulation of lncRNA Genes by Histone Modification in Alzheimer’s Disease. Biomed Res. Int..

[51] Zhao P., Liu S., Zhong Z., Jiang T., Weng R., Xie M., Yang S., Xia X. (2018). Analysis of expression profiles of long noncoding RNAs and mRNAs in brains of mice infected by rabies virus by RNA sequencing. Sci. Rep..

[52] Kim B.H., Shenoy A.R., Kumar P., Das R., Tiwari S., MacMicking J.D. (2011). A family of IFN-γ-inducible 65-kD GTPases protects against bacterial infection. Science.

[53] Osaki J.H., Espinha G., Magalhaes Y.T., Forti F.L. (2016). Modulation of RhoA GTPase Activity Sensitizes Human Cervix Carcinoma Cells to γ-Radiation by Attenuating DNA Repair Pathways. Oxidative Med. Cell Longev..

[54] Matos P. (2021). Small GTPases in Cancer: Still Signaling the Way. Cancers.

[55] Ma H., Qian W., Bambouskova M., Collins P.L., Porter S.I., Byrum A.K., Zhang R., Artyomov M., Oltz E.M., Mosammaparast N. (2020). Barrier-to-Autointegration Factor 1 Protects against a Basal cGAS-STING Response. mBio.

[56] Cheon H., Holvey-Bates E.G., McGrail D.J., Stark G.R. (2021). PD-L1 sustains chronic, cancer cell-intrinsic responses to type I interferon, enhancing resistance to DNA damage. Proc. Natl. Acad. Sci. USA.

[57] Hasipek M., Guan Y., Grabowski D., Gu X., Saunthararajah Y., Silverman R., Stark G., Maciejewski J., Jha B. (2020). Role of Oligoadenylate Synthetases in Myeloid Neoplasia. Blood.

