# Supplementary material for: Transcriptome Sequencing Reveals Tgf-β-Mediated Noncoding RNA Regulatory Mechanisms Involved in DNA Damage in the 661W Photoreceptor Cell Line
Source: Genes (Basel). 2022 Nov 17;13(11):2140. doi: 10.3390/genes13112140 (PMC9691224; doi:10.3390/genes13112140)
Supplement: Supplementary file 1 [file genes-13-02140-s001.zip › genes-1907944-supplementary.pdf]

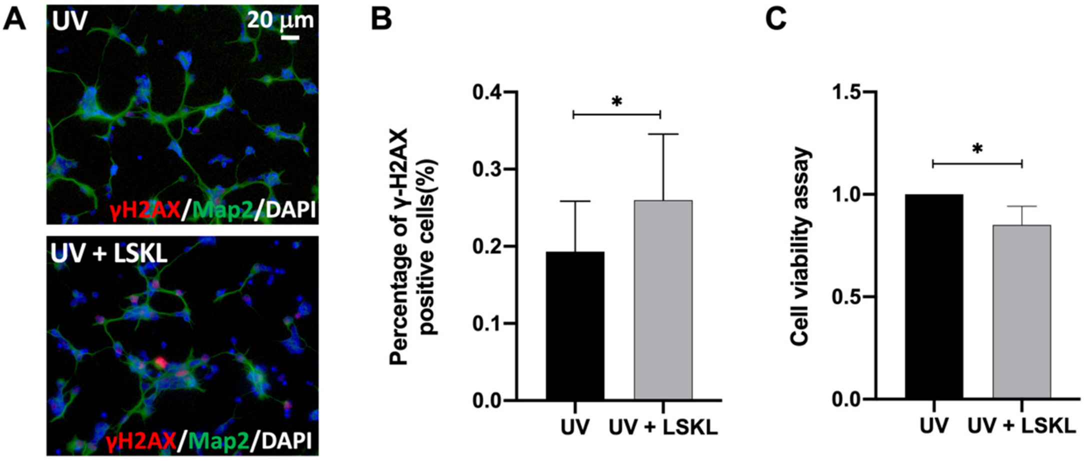

Figure S1. LSKL impaired DNA repair and cell viability in primary retinal neurons. Primary retinal neurons were pretreated with LSKL (24 h) or the control vehicle and exposed to UV irradiation. **(A)** The cells were then stained with γ-H2AX (red), Map2 (green) and DAPI (blue). Scale bars: 20 µm. **(B)** The percentage of γ-H2AX-positive primary retinal neurons was increased in the UV+LSKL group. **(C)** CCK8 assay showed that the cell viability of primary retinal neurons was impaired by LSKL pretreatment. *P<0.05.

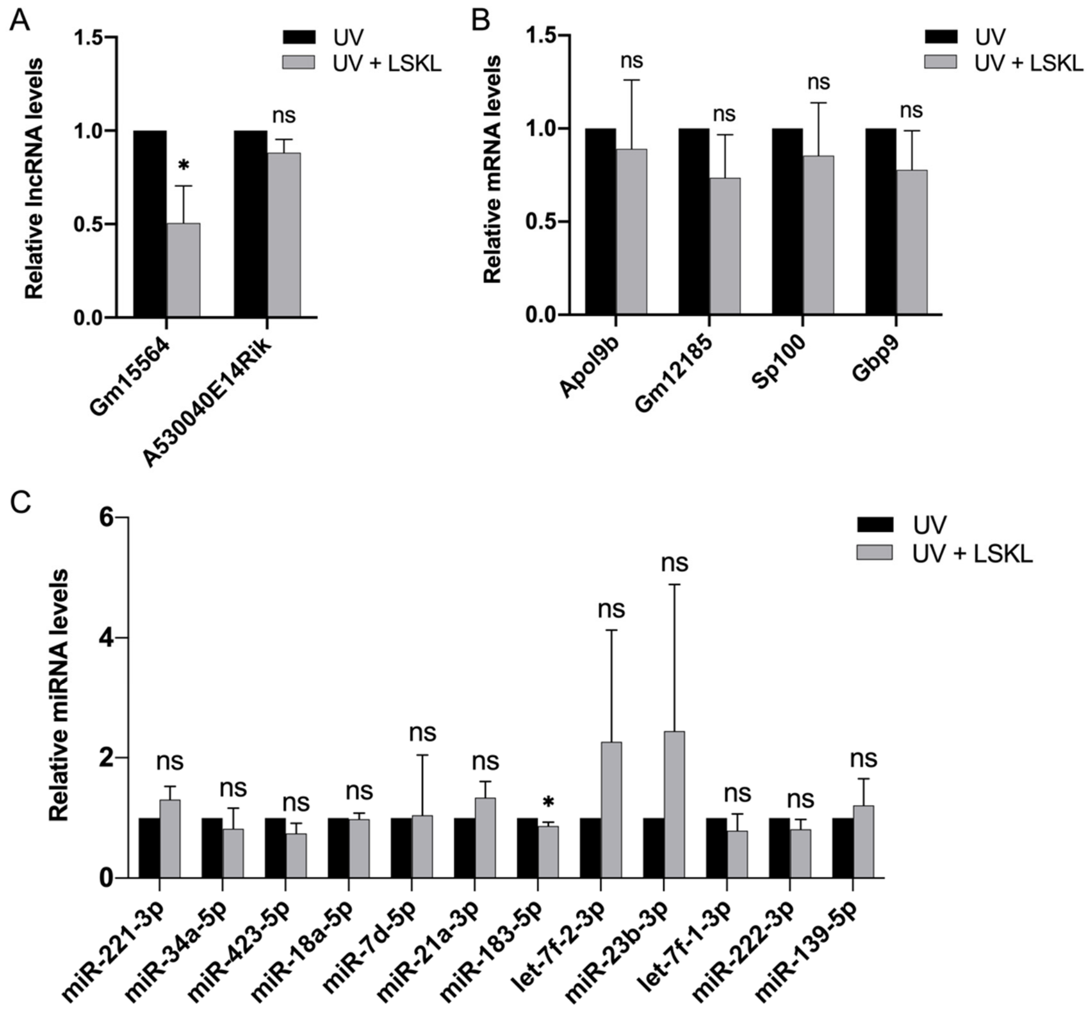

Figure S2. Relative RNA levels of lncRNA, miRNA, and mRNA components in the ceRNA network after LSKL treatment in 661W cells. 661W cells were pretreated with LSKL or control vehicle for 24 h and irradiated with UV. 1 h after UV exposure, the expression levels of lncRNAs **(A)**, mRNAs **(B)** and miRNAs **(C)** in UV- and UV+LSKL-treated 661W cells were detected by RT-qPCR. Data are presented as the mean ± SD from 3 independent experiments. *P<0.05. Ns, no significance.

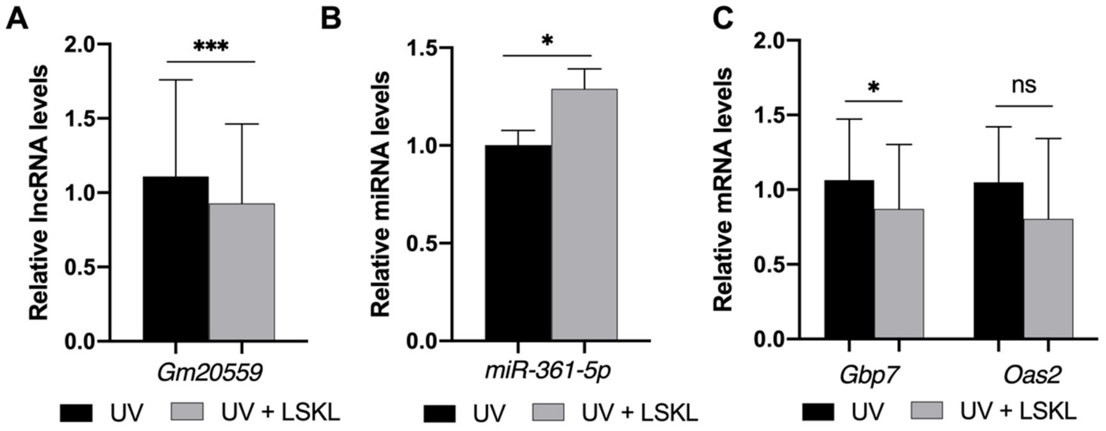

Figure S3. Relative RNA levels of lncRNA, miRNA, and mRNA components in the ceRNA network after LSKL treatment in primary retinal neurons. Primary retinal neurons were pretreated with LSKL or control vehicle for 24 h and irradiated with UV. 1 h after UV exposure, the expression levels of lncRNAs **(A)**, mRNAs **(B)** and miRNAs **(C)** in UV- and UV + LSKL-treated cells were detected by RT-qPCR. Data are presented as the mean ± SD from 3 independent experiments. *P<0.05, ***P<0.001. Ns, no significance.

Table S1: Primers used for RT-qPCR

Type	Name	Primer sequences	
		Forward 5'—3'	Reverse 5'—3'
mRNA	*Gbp5*	CAGACCTATTTGAACGCCAAAGA	TGCCTTGATTCTATCAGCCTCT
	Gbp7	AACTGAGGGTGAACTCCAAAGC	GTTTCAGACCTAACTGTGGTGC
	Sp100	TGATGGAGGGAACCCAAACTC	GGATGACCCATTCGGGCTT
	Pde1a	TTTCTTGCCTAATTGCCTTTGC	GAGCCAGTGCATGATACCTGT
	Apol9b	GGATACCTGTATAGCCTGGTGG	CAGTTCCCTCAGAGCTTTGTG
	Oas2	AAACCTCACACCCAACGAAAA	CCACCCTTAGCCACTTCCT
	Gbp9	TCGCCCTAAGTGTGCTTCTG	CGTGGGGATTTCGGGAAGA
	Gm12185	GGAGACCACCCTACTGAAGGA	GAAATCCCTCTTCCGGGCAA
	Actin	AGGTCATCACTATTGGCAACG	ACGGATGTCAACGTCACACTT
lncRNA	*Gm15564*	TCTCTCAGTCCCTACCTGCT	GTGCAGAGGAGGAGACAGAA
	Gm20559	GGTGGATTCAGCTCCGAGAG	GGCCATGATCTTACTTTGGCC
	A530040E14Rik	GCCCAGCTTAGGTGTGTCTA	CTCCAGGGATGAAGGGACAT
		RT 5'—3'	Forward 5'—3'
miRNA	miR-18a-5p	GTCGTATCCAGTGCGTGTCGTGGAGTCGGCAATTGCACTGGATACGAC**CTATCT**	GGGTAAGGTGCATCTAGTGC
	miR-27a-3p	GTCGTATCCAGTGCGTGTCGTGGAGTCGGCAATTGCACTGGATACGAC**GCGGAA**	GGGGTTCACAGTGGCTAAG
	let-7f-2-3p	GTCGTATCCAGTGCGTGTCGTGGAGTCGGCAATTGCACTGGATACGAC**GAAAGA**	GGGCGCTATACAGTCTACTG
	let-7f-1-3p	GTCGTATCCAGTGCGTGTCGTGGAGTCGGCAATTGCACTGGATACGAC**GGGAAG**	GGGCGCTATACAATCTATTGC
	miR-221-3p	GTCGTATCCAGTGCGTGTCGTGGAGTCGGCAATTGCACTGGATACGAC**GAAACC**	GGGAGCTACATTGTCTGCTG
	miR-222-3p	GTCGTATCCAGTGCGTGTCGTGGAGTCGGCAATTGCACTGGATACGAC**ACCCAG**	GGGGAGCTACATCTGGCTA
	miR-139-5p	GTCGTATCCAGTGCGTGTCGTGGAGTCGGCAATTGCACTGGATACGAC**CTGGAG**	GGGTCTACAGTGCACGTGT
	miR-23b-3p	GTCGTATCCAGTGCGTGTCGTGGAGTCGGCAATTGCACTGGATACGAC**GGTAAT**	GGGATCACATTGCCAGGG
	let-7d-5p	GTCGTATCCAGTGCGTGTCGTGGAGTCGGCAATTGCACTGGATACGAC**AACTAT**	GGGGAGAGGTAGTAGGTTGC
	miR-361-5p	GTCGTATCCAGTGCGTGTCGTGGAGTCGGCAATTGCACTGGATACGAC**GTACCC**	GGGGGTTATCAGAATCTCCAG
	miR-423-5p	GTCGTATCCAGTGCGTGTCGTGGAGTCGGCAATTGCACTGGATACGAC**AAAGTC**	GGATGAGGGGCAGAGAGCGA
	miR-34a-5p	GTCGTATCCAGTGCGTGTCGTGGAGTCGGCAATTGCACTGGATACGAC**ACAACC**	GGGTGGCAGTGTCTTAGCT
	miR-21a-3p	GTCGTATCCAGTGCGTGTCGTGGAGTCGGCAATTGCACTGGATACGAC**GACAGC**	GGGCAACAGCAGTCGATGG
	miR-183-5p	GTCGTATCCAGTGCGTGTCGTGGAGTCGGCAATTGCACTGGATACGAC**AGTGAA**	GGGGTATGGCACTGGTAGAA
	Loop-Reverse	CAGTGCGTGTCGTGGAGT	
	U6	CGCTTCACGAATTTGCGTGTCAT	GCTTCGGCAGCACATATACTAAAAT

Table S2: Sequences of siRNAs and miRNA mimics

Type	Name	Sequences	
		Forward 5'—3'	Reverse 5'—3'
siRNA	*Gm20559* #1	GUACAGGUCUAUCGAAUUATT	UAAUUCGAUAGACCUGUACTT
	Gm20559 #2	GACUACUCCUCCAAUAGAATT	UUCUAUUGGAGGAGUAGUCTT
	Gm20559 #3	CCAGCAAAUUGUCACCUAATT	UUAGGUGACAAUUUGCUGGTT
miRNA mimics	miR-361-5p	UUCACAGUGGCUAAGUUCCGC	GGAACUUAGCCACUGUGAAUU
